# Altered Metabolites in the Plasma of Autism Spectrum Disorder: A Capillary Electrophoresis Time-of-Flight Mass Spectroscopy Study

**DOI:** 10.1371/journal.pone.0073814

**Published:** 2013-09-18

**Authors:** Hitoshi Kuwabara, Hidenori Yamasue, Shinsuke Koike, Hideyuki Inoue, Yuki Kawakubo, Miho Kuroda, Yosuke Takano, Norichika Iwashiro, Tatsunobu Natsubori, Yuta Aoki, Yukiko Kano, Kiyoto Kasai

**Affiliations:** 1 Department of Child Neuropsychiatry, Graduate School of Medicine, The University of Tokyo, Bunkyo-ku, Tokyo, Japan; 2 Department of Neuropsychiatry, Graduate School of Medicine, The University of Tokyo, Bunkyo-ku, Tokyo, Japan; 3 Japan Science and Technology Agency, CREST, Chiyoda-ku, Tokyo, Japan; 4 Office for Mental Health Support, Division for Counseling and Support, The University of Tokyo, Bunkyo-ku, Tokyo, Japan; 5 Department of Psychology, Faculty of Integrated Human and Social Welfare, Shukutoku University, Chiba, Japan; Chiba University Center for Forensic Mental Health, Japan

## Abstract

Clinical diagnosis and severity of autism spectrum disorders (ASD) are determined by trained clinicians based on clinical evaluations of observed behaviors. As such, this approach is inevitably dependent on the expertise and subjective assessment of those administering the clinical evaluations. There is a need to identify objective biological markers associated with diagnosis or clinical severity of the disorder. To identify novel candidate metabolites as potential biomarkers for ASD, the current study applied capillary electrophoresis time-of-flight mass spectroscopy (CE-TOFMS) for high-throughput profiling of metabolite levels in the plasma of 25 psychotropic-naïve adult males with high-functioning ASD and 28 age-matched typically-developed control subjects. Ten ASD participants and ten age-matched controls were assigned in the first exploration set, while 15 ASD participants and 18 controls were included in the second replication set. By CE-TOFMS analysis, a total of 143 metabolites were detected in the plasma of the first set. Of these, 17 metabolites showed significantly different relative areas between the ASD participants and the controls (*p*<0.05). Of the 17 metabolites, we consistently found that the ASD participants had significantly high plasma levels of arginine (*p* = 0.024) and taurine (*p* = 0.018), and significantly low levels of 5-oxoproline (*p*<0.001) and lactic acid (*p* = 0.031) compared with the controls in the second sample set. Further confirmatory analysis using quantification of absolute metabolite concentrations supported the robustness of high arginine (*p* = 0.001) and low lactic acid (*p* = 0.003) in the combined sample (n = 53). The present study identified deviated plasma metabolite levels associated with oxidative stress and mitochondrial dysfunction in individuals with ASD.

## Introduction

Autism spectrum disorder (ASD) is a neurodevelopmental disorder characterized by social deficits, communication deficits and restricted and repetitive behaviors [1]. Diagnosis or clinical severity of ASD is mostly determined by trained clinicians based on clinical evaluations of observed behaviors [2], [3]. As such, this approach is inevitably dependent on the expertise of those administering the diagnostic tests [4]. Thus, more objective methodologies for evaluating diagnosis or clinical severity of the disorder has been sought after [5]–[9]. These studies have revealed a higher mRNA expression in lymphocytes [5], metabolite alterations in the hippocampal formation [7], atypical brain responses to social stimuli [8], and increased serum levels of anterior pituitary hormones [9]. However, to the best of our knowledge, there are no reliable and practical biological markers for assessing clinical diagnosis or severity of ASD.

Metabolomics is a high-throughput metabolic profiling method to evaluate the concentration of metabolites in a given sample, such as plasma and urine. Because the status of metabolites is considered to be affected by pathophysiological processes, this approach can identify candidate molecules for representing the pathophysiology of developmental and psychiatric disorders [10]–[12]. To date, previous metabolomics studies using nuclear magnetic resonance (NMR), liquid chromatography mass spectroscopy (LC-MS), and gas chromatography MS (GC-MS) have reported possible biomarkers, such as amino acids, antioxidants, gut bacterial metabolites, and sulfur in urine from children with ASD [13], [14]. Although urine collection is totally non-invasive and holds promise for the disease biomarkers, a portion of the plasma metabolites are biotransformed in the kidneys before production of urine [4], [15]. Actually, different candidate biological markers for other disorders have been detected from urine and plasma samples [15], [16]. Previous literature suggests that changes in potential biomarkers in cerebrospinal fluid might be correlated with those in plasma [6]. However, to our knowledge, there was no previous study reporting metabolomic profiles quantified from plasma in individuals with ASD.

Capillary electrophoresis time-of-flight mass spectroscopy (CE-TOFMS) is a relatively new MS method that can measure metabolites of higher ionization and lower molecular weight with easy preparation and high throughput [17], [18]. While the efficacy of CE–TOFMS has been demonstrated in various human clinical studies [19], [20], no previous application of this method for ASD subjects has been reported.

To identify novel candidate metabolites as potential biomarkers for ASD, the current study applied CE-TOFMS to plasma samples from subjects with ASD and age-matched typically-developed control subjects. To minimize potential confounding effects of medications and neuropsychiatric comorbidities, including mental retardation and epilepsy, on metabolite levels, the participants with ASD, as well as those with typical development, were confined to psychotropic naïve subjects and free from neuropsychiatric comorbidities.

## Methods

### Ethics Statement

All the participants provided written informed consent after they were given a complete explanation of the study as approved by the ethics committee of The University of Tokyo Hospital (No. 2094-[5]). Using longitudinal clinical assessments, a trained psychiatrist (H.Y.) confirmed that all of these adult participants had no intellectual disabilities and no need for psychotropic medication, and were capable of providing informed consent. Therefore, no participant needed for someone else to provide consent on their behalf.

### Study Participants

A total of 53 Japanese adults (all Asian ethnicity) participated in this study. Considering the relatively low analytical reproducibility of capillary electrophoresis [11], we randomly enrolled two independent sets of samples for exploration and replication. The participants were recruited from April 2010 to July 2012 at the outpatient clinic of The University of Tokyo Hospital. All the study participants were male, since previous studies have indicated the potential contribution of sexual dimorphism to the pathophysiology of ASD [21], [22]. Ten ASD participants and ten age-matched controls were assigned in the first set (exploration set), while 15 ASD participants and 18 age-matched control subjects were included in the second set (replication set) (Table 1). All the ASD participants were psychotropic-naïve. The diagnostic protocols and clinical assessments were described detail in our previous papers [23], [24]. Briefly, an experienced psychiatrist (H.Y.) carefully diagnosed the participants with ASD on the basis of DSM-IV-TR [1] criteria after more than 2 months of follow-up examinations. Another trained psychiatrist (H.K.) confirmed the clinical diagnoses of the ASD participants using the Japanese version of the Autism Diagnostic Interview–Revised (ADI-R) [3], [25]. For the six participants who did not meet the threshold in the ADI-R social domain, the ASD diagnosis was confirmed using the Autism Diagnostic Observation Schedule - Generic [2] by a trained psychologist (M.K.). Moreover the Global Assessment of Functioning (GAF) [1] was evaluated in all the ASD participants. The verbal Intelligence quotients (IQ) of the control group (all the participants in the first set and eight participants in the second set) was estimated using a Japanese version of the National Adult Reading Test [26], [27]. Although the National Adult Reading Test can measure IQ accurately in controls, the test is problematic for ASD participants because of the well-known imbalances in their intellectual abilities. Therefore, the IQs of the ASD participants were assessed using the Wechsler Adult Intelligent Scale-revised [28] or Wechsler Adult Intelligent Scale third edition [29].

**Table 1:** Demographic characteristics of the study participants.

	First sample set		Second sample set	
	ASD (n = 10)	Controls (n = 10)	ASD (n = 15)	Controls (n = 18)
Age (years)	32.2±7.0	32.9±3.6	28.6±5.3	28.7±4.0
ADI-R S	12.3±5.4	NA	16.9±6.8	NA
ADI-R C	9.5±2.0	NA	12.8±4.6	NA
ADI-R R	3.3±2.0	NA	4.8±1.8	NA
GAF	45.5±8.8	NA	46.7±6.8	NA
Full Scale IQ	102.5±11.2	NA	109.3±9.5	NA
Verbal IQ	111.3±11.7	115.2±4.7	113.0±12.9	108.8±9.3
Performance IQ	90.3±14.1	NA	100.9±17.0	NA

Values are given in mean ± SD, except for the number of participants.

Abbreviations: ASD, autism spectrum disorders; ADI-R, autism diagnostic interview revised; S, social domain; C, communication domain; R, restricted and repetitive behavior domain; GAF, global assessment of functioning; NA, not applicable.

The exclusion criteria were: neurological illness, traumatic brain injury with any known cognitive consequences or loss of consciousness for more than 5 min, low IQ (i.e. below 80), previous alcohol dependence, previous continuous illegal substance use (e.g., cannabis). Additional exclusion criteria for the control group were any past and present psychiatric disease detected by screening with the modified Mini-International Neuropsychiatric Interview [30] or a family history of axis I disorder within the first-degree relatives.

### CE-TOFMS Analysis

Peripheral blood samples were drawn by experienced physicians from a peripheral vein while the participant was fasting (>3 h without any meal and/or nutritious drink). Plasma samples were isolated via centrifugation at 1200 g for 10 min, and then stored at −80°C until use.

Plasma samples (100 µL) were immersed into 0.45 mL methanol containing 10 µM each methionine sulfone and 10-camphorsulfonic acid, and mixed well. Then, 200 µL deionized water and 0.5 mL chloroform were added and the solution was centrifuged at 2,300 g for 5 min at 4°C. The upper aqueous layer was centrifugally filtered through a 5-kDa cutoff filter (Human Metabolome Technologies Inc., Tsuruoka, Japan) to remove proteins. The filtrate was lyophilized and dissolved in 50 µL of ultrapure water containing reference compounds prior to mass spectrometry analysis. The water was produced by a Milli-Q Academic A10 (EMD Millipore, Billerica, MA, USA).

Samples were applied to a capillary electrophoresis system equipped with an Agilent 6210 time-of-flight mass spectrometer (CE-TOFMS, Agilent Technologies, Santa Clara, CA, USA), as previously described [31]. Raw data files from CE-TOFMS were processed using customized proprietary software written in Java (An extended version of MathDAMP that has been developed in Keio University) [32]. The software performs (1) peak picking, and (2) peak alignment. For (1), all peaks potentially corresponding to metabolites were extracted. After peak picking, the migration time of electrophoresis was normalized using those of the internal standards. For (2), an alignment was applied according to similar mass-to-charge ratios (m/z) and normalized migration times. The tolerance was set to 100 ppm (m/z) and 0.5 min (Normalized migration time). The peak matrix was matched with the annotation table of the metabolomics library (The Basic Scan metabolomics service of Human Metabolome Technologies Inc.) described previously based on their m/z and migration times [33]. All peak areas were divided by the area of the internal standard (i.e. Relative area) to normalize the signal intensities, and to avoid injection-volume bias and mass-spectrometry detector sensitivity bias among multiple measurements.

### Statistical Analysis

#### Comparisons for relative metabolite concentrations between individuals with ASD and controls

All statistical analyses were conducted using the SPSS version 17.0 (IBM Inc., Armonk, NY, USA). As the current sample sizes were relatively small, normal distributions of the present data sets across all metabolites were not warranted in advance. Therefore, non-parametric statistics were employed for the statistical analyses. The obtained relative area values of each metabolite were regarded as their relative concentration. We tested the differences of these relative concentrations between the ASD participants and the controls using two-tailed Mann-Whitney U test in the first set. For the purpose of exploration, significance levels were set at *p*<0.05 for each metabolite without correction for multiple comparisons. For the purpose of replication, we compared the metabolite levels, which showed significant differences due to diagnosis in the first set, between the participants with ASD and the controls in the second set using one-tailed Mann-Whitney U test. As analyses on the second set were aimed to replicate findings in the first set, the directions of expected effects were known *a priori*. Thus, one-tailed tests (*p*<0.05 were set as significance) were used without correction for multiple comparisons [34].

#### Discriminant analyses between ASD and controls based on the identified metabolites

To preliminarily test the possibility of the detected metabolites as a diagnostic tool, we conducted discriminant analyses using metabolite levels that were significantly and consistently different in the ASD participants in the first and the second sets. Since the discriminant analyses were conducted separately on the first and second sets, the significance levels were set at *p*<0.025 with Bonferroni correction for two data sets.

#### Comparisons for absolute metabolites concentrations between ASD individuals and controls

Analysis by CE-TOFMS in our system enables measurement of the absolute quantities of pre-determined 108 major metabolites, based on the peak area of internal controls of each metabolite. Since the quantity of these metabolites can be reliably compared across different experimental batches, absolute concentrations were quantified for these metabolites for the purpose of further verification in the combined sample of the first and second sets. We compared absolute concentrations of the major metabolites using two-tailed U test between the 25 ASD individuals and 28 controls. To test the robustness of findings from comparisons using relative metabolite measures, statistical significance was set at *p*<0.05/N (number of metabolites absolutely quantified among the metabolites consistently showed significant differences at relative concentrations in both the first and second sets) employing Bonferroni correction.

#### Correlation analyses between metabolites and clinical measures

To explore clinical significance of the deviated metabolites in the plasma from ASD subjects, we calculated Spearman’s rank correlation coefficients between their clinical characteristics (i.e. ADI-R, GAF, IQ) and absolute value of significant metabolites in the combined sample set. Since the correlation analyses were implicated as exploratory analyses, we adopted absolute values in the combined sample set to increase the statistical power, and then statistical significance was defined as *p*<0.05.

## Results

**Figure 1:**
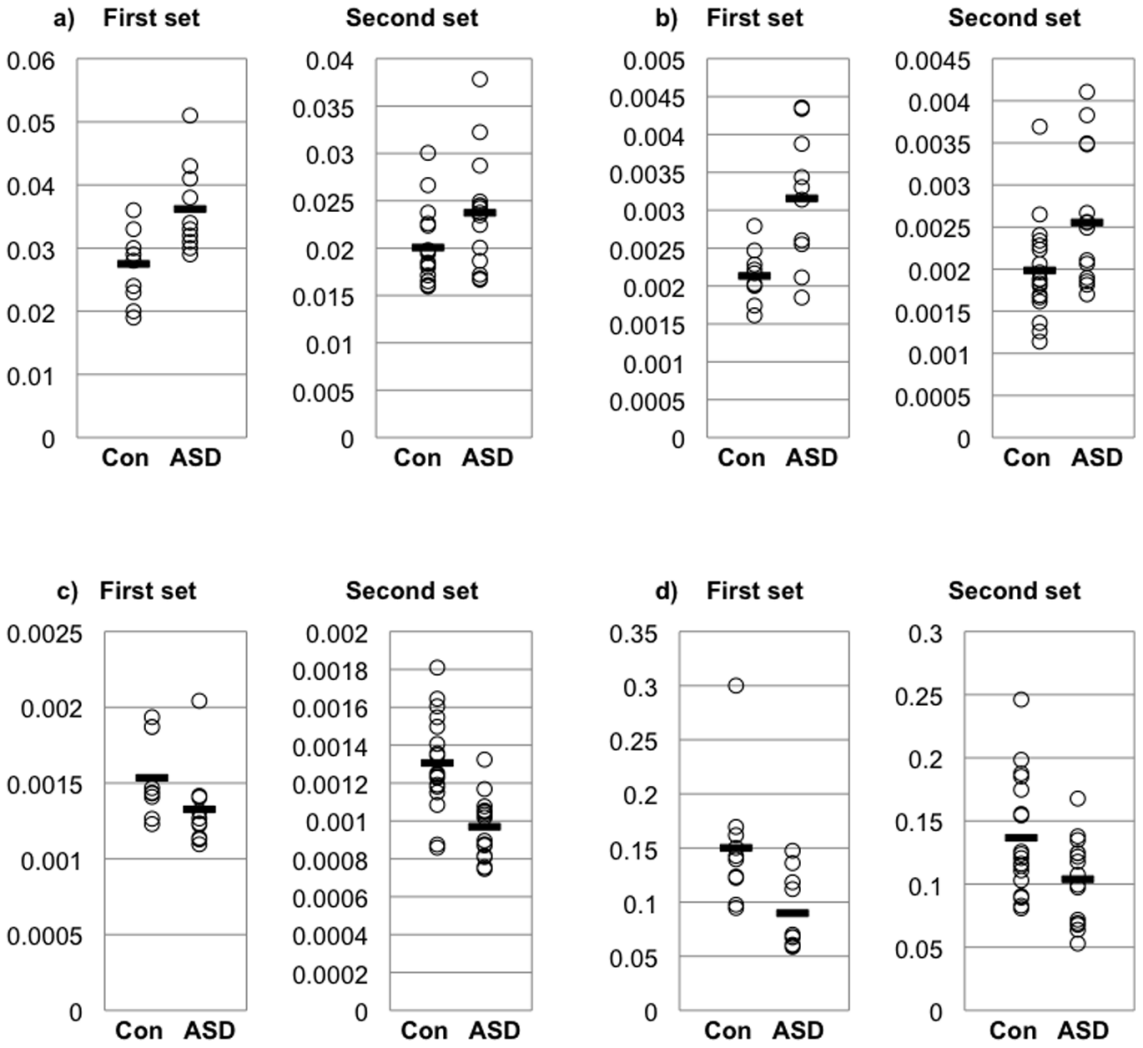
Metabolites with significantly deviated relative concentrations in the ASD participants. Plots show relative areas of arginine (a), taurine (b), 5-oxoproline (c), lactic acid (d), in the first (left) and the second (right) sample sets. Y-axis indicates relative concentrations. Bars indicate mean concentration in the group. Con, controls; ASD, participants with autistic spectrum disorders.

By the CE-TOFMS analysis, a total of 143 metabolites were detected in the plasma samples of the first sample set (79 and 64 metabolites for the cation and anion modes, respectively). Of these, 17 metabolites showed significantly different relative areas between the ASD participants and the controls. Of the 17 metabolites which showed significant difference in the first sample set, 14 metabolites were successfully quantified in the second sample set, although a total of 141 metabolites were measurable (76 and 65 metabolites for cation and anion modes, respectively). The lists of detected metabolites in the first and the second sample sets were available in Table S1. For the purpose of validating the findings in the first sample set, we conducted a one-tailed Mann-Whitney U test for the relative areas of the 14 metabolites. Then, we found that the ASD participants had significantly high levels of arginine (*p* = 0.024) and taurine (*p* = 0.018), low levels of 5-oxoproline (*p*<0.001) and lactic acid (*p* = 0.031) compared with the controls in the second sample set (Table 2 and Figure 1).

**Table 2:** Results from comparisons for relative metabolite concentrations between individuals with ASD and controls.

Direction of change					First sample set^a^			Second sample set^a^		
	Metabolites	Mode	m/z	MT (min)	ASD	Controls	p value^b^	ASD	Controls	p value^c^
Higher	Arginine	Cation	175.12	7.12	0.036 (0.0070)	0.028 (0.0060)	0.011	0.024 (0.0059)	0.020 (0.0037)	0.024
	Taurine	Cation	126.02	23.31	0.0031 (0.00088)	0.0021 (0.00034)	0.007	0.0026 (0.00081)	0.0020 (0.00059)	0.018
Lower	5-Oxoproline	Anion	128.03	9.50	0.0013 (0.00027)	0.0015 (0.00026)	0.035	0.00097 (0.00016)	0.0013 (0.00025)	<0.001
	Lactic acid	Anion	89.02	10.93	0.090 (0.035)	0.15 (0.058)	0.009	0.10 (0.033)	0.14 (0.046)	0.031

a Mean relative area value and its SD are given.

b Two- and ^c^one-tailed Mann-Whitney U tests.

Abbreviations: m/z, mass-to-charge ratio; MT, migration time; ASD, participants with autism spectrum disorder; NA, not applicable; ND not detected.

**Figure 2:**
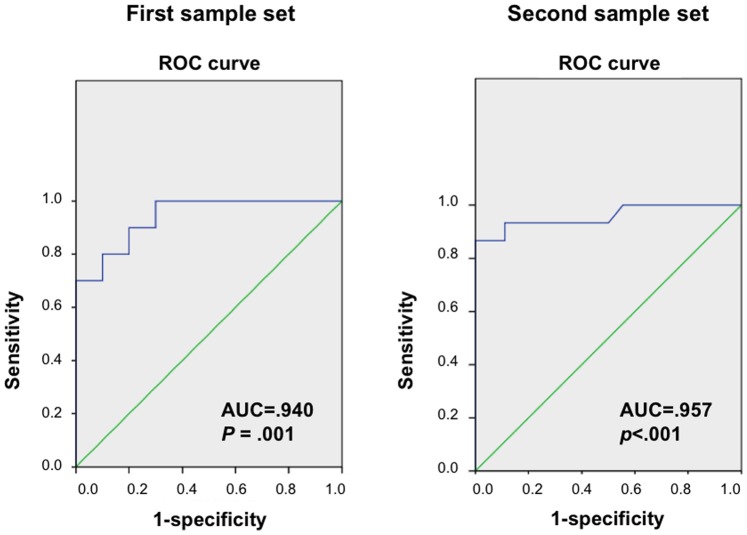
Results of discriminant analyses. A receiver-operating characteristic (ROC) curve and area under the curve (AUC) according to the results of discrimination analysis in the first (left) and the second sample sets (right) are presented. The results of discriminant analyses between the subjects with autism spectrum disorders and controls are indicated with blue lines.

Discriminant analysis in the first sample set showed that diagnosis of 80.0% (16 out of 20) of the subjects was correctly classified with the four used metabolites (arginine, taurine, 5-oxoproline, and lactic acid; Wilks’s *λ* = 0.447, *p*<0.012, Figure 2). These four metabolites were differentiated by participants with ASD and controls with an area under the receiver-operating characteristic curve (AUC) value of 0.940 (*p* = 0.001). These discriminant analyses in the second sample set showed that diagnosis of 78.8% (26 out of 33) of the subjects was correctly classified (*λ* = 0.437, *p*<0.001, Figure 2), and AUC value was 0.957 (*p*<0.001).

Among pre-determined 108 major metabolites, which could measure absolute quantities, 39 metabolites were identified in all the 53 participants (25 ASD participants and 28 controls). Among the four metabolites listed in Table 2, two metabolites (arginine, lactic acid) were included in the 39 major metabolites identified. We thus compared their absolute concentrations using the combined dataset. The significance level was defined at *p*<0.025 with Bonferroni correction for two metabolites. Absolute concentration of arginine was significantly higher (*p* = 0.001), while that of lactic acid was significantly lower (*p* = 0.003) in the ASD group than in the controls (Table 3).

**Table 3:** Absolute quantities of the metabolites in the ASD and control groups.

	Concentration (µM)^a^		
Metabolites	ASD	Controls	p value^b^
Arginine	96.3 (21.6)	78.3 (15.2)	0.001
Lactic acid	1678.3 (661.6)	2406.0 (868.3)	0.003

a Mean concentration and its SD are given. ^b^Two-tailed Mann-Whitney U tests. Abbreviation: ASD, participants with autism spectrum disorders.

**Figure 3:**
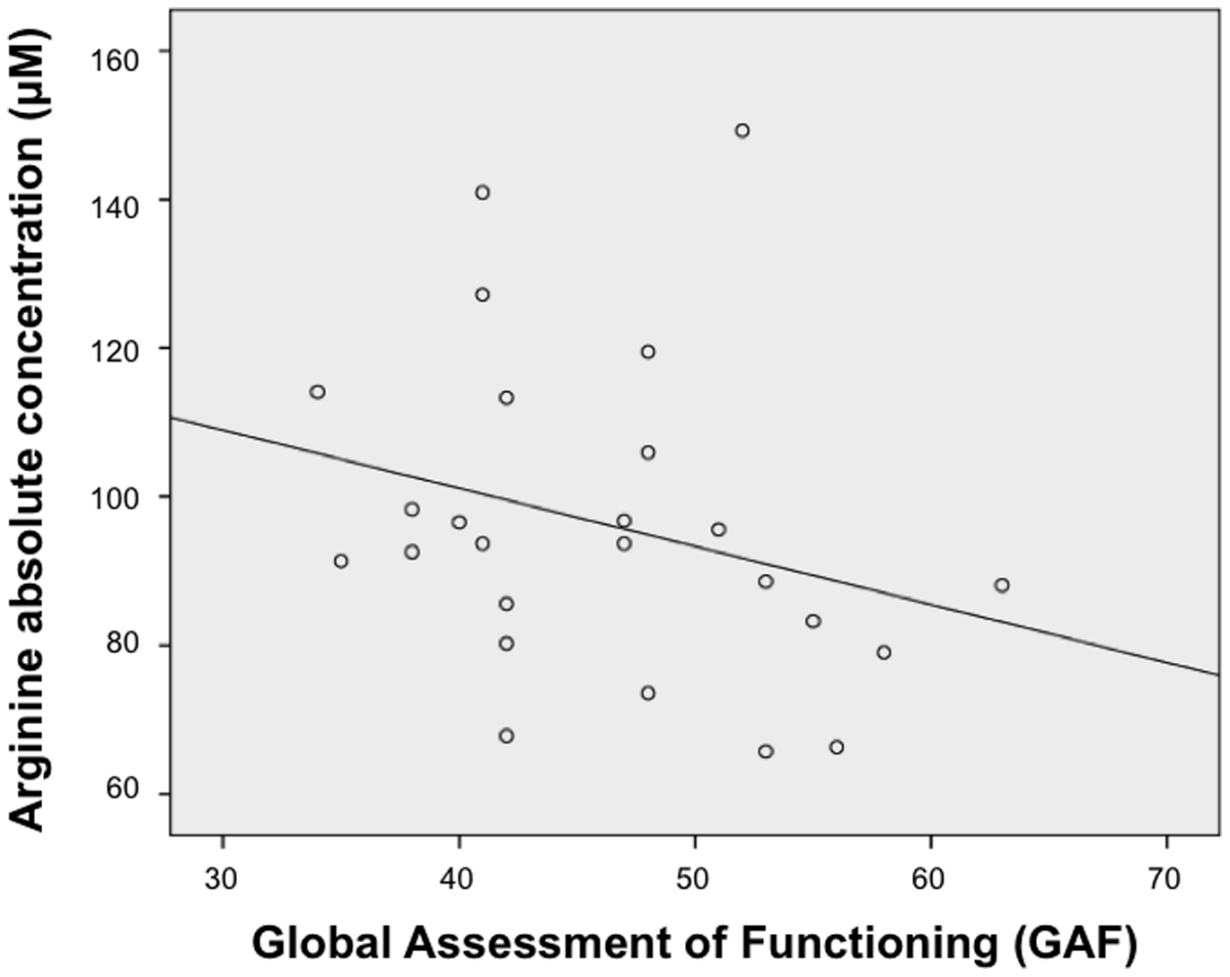
Relationship between arginine absolute concentration and GAF score. Scatter plot shows correlation coefficients between arginine absolute concentration and GAF score (*rho* = −0.413, *p* = 0.040).

Correlation analysis using Spearman’s rank correlation coefficients in the combined sample showed significant negative correlation between the concentration of arginine and GAF score (rho = −0.413, *p* = 0.040, Figure 3). There was no significant correlation between metabolites and ADI-R or IQ scores.

## Discussion

The current metabolomic analysis using CE-TOFMS revealed that plasma levels of several metabolites were significantly different between the participants with ASD and controls. We analyzed two independent sample sets, and consistently identified high levels of arginine and taurine, and low levels of lactic acid and 5-oxoproline in the participants with ASD compared with the controls. Taking advantage of the function of absolute concentration quantification of major metabolites, altered concentrations of arginine and lactic acid in the participants with ASD were further confirmed. To the best of our knowledge, this is the first study that elucidated possible biomarkers in peripheral blood plasma to evaluate ASD using metabolomics analysis.

Arginine is an essential precursor for the synthesis of proteins and other molecules of enormous biological importance, including nitric oxide (NO). Excessive arginine is thought to induce oxidative stress via NO production [35]. Though the present CE-TOFMS analyses were not able to measure nitric oxide concentration, NO level was previously reported to be high in ASD children [36]. However, children with ASD were reported to have no difference in plasma arginine concentration [37], and there is no report that examined arginine concentration in adults with ASD other than the present study. On the other hand, research on other psychiatric disorders, such as schizophrenia, have reported excess [38] or reduced plasma arginine levels [12]. The precise nature of the relationship between arginine and oxidative stress in neuropsychiatric disorders is unclear; however, the common susceptibility genes for ASD and schizophrenia, TCF4 [39] and NOS1 [40], [41] has been suggested to be involved in the arginine-NO pathway. The arginine concentration was negatively correlated with GAF score in the current participants with ASD, although there was no significant correlation with ADI-R scores. Therefore, the current study could not draw the conclusion that arginine was a specific biomarker for the pathogenesis of ASD rather than that for general difficulties due to psychiatric disorders. Besides, since ADI-R scores are calculated mainly based on behavioral characteristics during childhood, a possibility that arginine concentration reflect the current ASD severity during adulthood rather than that during childhood remains. Future studies should address these issues.

The inhibitory amino acid taurine is an osmoregulator and neuromodulator, also exerting protective actions in neural tissue, including under conditions of oxidative stress [42]. Plasma taurine concentration in children with ASD was reported to be high compared with typically developing children [37], [43], and other researches has reported contradictory results that showed low concentrations of taurine in ASD children [44]–[46]. Similarly, contradictory changes in urine concentrations of taurine in ASD children were reported, with elevated [13] and low concentrations [14]. Although the reason for the contradictory findings in plasma and urine concentrations remains unclear, taurine is hypothesized to be protective for autistic symptoms [47], [48]. The present result, which showed elevated plasma taurine level in adults with ASD, might be due to compensatory regulation against pathogenesis of ASD, such as oxidative stress.

The endogenous molecule 5-oxoproline is derived from L-glutamate, being a major intermediate in the γ-glutamyl cycle. This cycle is necessary for the synthesis and breakdown of glutathione, and also for the intracellular transport of free amino acids [49]. Although the pathological role of 5-oxoproline in the human brain is not clarified, it has been presumed from animal studies that 5-oxoproline elicits oxidative stress, which may represent a pathophysiological mechanism in the neuro-pathological disorder in which this metabolite accumulates [50], [51]. Direct contribution of 5-oxoproline down-regulation in ASD pathology is not clear, but like taurine and arginine, 5-oxoproline might be a putative biomarker for ASD that has oxidative stress alterations.

Lactic acid is thought to be one of the biochemical markers that may indicate mitochondrial function [52]. Previous studies have suggested that mitochondrial dysfunction and altered energy metabolism may influence the social and cognitive deficits in autism [53]–[56]. A school-based study of 69 children aged 11 to 14 years with ASD found mitochondrial respiratory chain dysfunction and hyperlactacidemia [57]. The present results showed that the lower lactic acid concentration in ASD participants was contradictory to the results of Oliveira et al. (2005). A possible explanation for this inconsistency is that the present sample sets consisted of adults with ASD. In conjunction with the reported high lactic acid concentration in children with ASD, low lactic acid concentration in adults with ASD in the present results might suggest altered regulation of mitochondrial function during development. There was no significant correlation between lactic acid level and clinical measures, therefore further studies were needed to scrutinize clinical significance of low lactic acid concentration in adults with ASD.

It should be noted that this study has several potential methodological limitations. First, the present study consisted of male subjects only. Though we were able to avoid confounding sex influence on the results, the present findings might not be generalizable to females. Second, longitudinal metabolite measurements with detailed clinical investigations from childhood will be needed to determine if the identified metabolites showed development-dependent changes. Third, the current discriminant analyses successfully classified the participants with ASD and controls using concentrations of the detected four metabolites. However, since the current discriminant analyses used the same sample sets as those for detection of the four metabolites owing to the limited sample size of the present study, the possibility for discrimination remains to be validated in a future study with independent samples. In addition, other psychiatric disorders (e.g. schizophrenia) have to be considered as a clinical control group in future studies, because the pathophysiology of these psychiatric disorders is thought to be shared [58], [59]. Comparison among other psychiatric disorders may clarify the pathogenesis more clearly. Fourth, CE-TOFMS is not very effective for the separation of neutral metabolites and large molecules. Concomitant analysis of the same sample by LC-MS, GC-MS, and NMR approaches has the potential to greatly expand the coverage of target metabolites. Finally, the total sample size of the present study (n = 53) was relatively small, although, by conducting power analyses based on the effect sizes of the four altered metabolites detected in the current study (d = 0.72–1.24), the present study provides useful information about required total sample sizes in future studies (n = 58–158) to detect the deviated metabolites with more conservative threshold for statistical significance with power of 80% (e.g. Bonferroni correction).

In conclusion, the present study measured small ionic metabolites in peripheral blood plasma using the CE-TOFMS system, and found elevated and reduced metabolites in plasma samples from ASD participants associated with markers of oxidative stress and mitochondrial dysfunction. These metabolites could become possible biomarkers for differential diagnosis, determination of severity, and prediction of drug response, which could promote better treatment options and result in better prognosis.

## Supporting Information

Table S1 **Detected metabolites list.** Metabolites detected in both the first and second sets, those in the first set only, and those in the second set only were listed respectively.
(DOCX)

## References

[1] American Psychiatric Association (2000) Diagnostic and Statistical Manual of Mental Disorders. Fourth edition. Text revision. Washington DC: American Psychiatric Association.

[2] Lord C, Risi S, Lambrecht L, Cook EH Jr, Leventhal BL, et al (2000) The autism diagnostic observation schedule-generic: a standard measure of social and communication deficits associated with the spectrum of autism. J Autism Dev Disord 30: 205–223. 11055457

[3] Tsuchiya KJ, Matsumoto K, Yagi A, Inada N, Kuroda M, et al (2013) Reliability and validity of autism diagnostic interview-revised, Japanese version. J Autism Dev Disord 43: 643–662. 22806002 10.1007/s10803-012-1606-9

[4] Emond P, Mavel S, Aidoud N, Nadal-Desbarats L, Montigny F, et al (2013) GC-MS-based urine metabolic profiling of autism spectrum disorders. Anal Bioanal Chem 405: 5291–5300. 23571465 10.1007/s00216-013-6934-x

[5] Nakamura K, Anitha A, Yamada K, Tsujii M, Iwayama Y, et al (2008) Genetic and expression analyses reveal elevated expression of syntaxin 1A (STX1A) in high functioning autism. Int J Neuropsychopharmacol 11: 1073–1084. 18593506 10.1017/S1461145708009036

[6] Quinones MP, Kaddurah-Daouk R (2009) Metabolomics tools for identifying biomarkers for neuropsychiatric diseases. Neurobiol Dis 35: 165–176. 19303440 10.1016/j.nbd.2009.02.019

[7] Suzuki K, Nishimura K, Sugihara G, Nakamura K, Tsuchiya KJ, et al (2010) Metabolite alterations in the hippocampus of high-functioning adult subjects with autism. Int J Neuropsychopharmacol 13: 529–534. 19895725 10.1017/S1461145709990952

[8] Kaiser MD, Hudac CM, Shultz S, Lee SM, Cheung C, et al (2010) Neural signatures of autism. Proc Natl Acad Sci U S A 107: 21223–21228. 21078973 10.1073/pnas.1010412107 PMC3000300

[9] Iwata K, Matsuzaki H, Miyachi T, Shimmura C, Suda S, et al (2011) Investigation of the serum levels of anterior pituitary hormones in male children with autism. Mol Autism 2: 16. 22011527 10.1186/2040-2392-2-16 PMC3215952

[10] Zheng P, Gao HC, Li Q, Shao WH, Zhang ML, et al (2012) Plasma metabonomics as a novel diagnostic approach for major depressive disorder. J Proteome Res 11: 1741–1748. 22239730 10.1021/pr2010082

[11] Xu XH, Huang Y, Wang G, Chen SD (2012) Metabolomics: a novel approach to identify potential diagnostic biomarkers and pathogenesis in Alzheimer’s disease. Neurosci Bull 28: 641–648. 23054640 10.1007/s12264-012-1272-0 PMC5561924

[12] Pilling S, Baron-Cohen S, Megnin-Viggars O, Lee R, Taylor C (2012) Recognition, referral, diagnosis, and management of adults with autism: summary of NICE guidance. BMJ 344: e4082. 22740567 10.1136/bmj.e4082

[13] Yap IK, Angley M, Veselkov KA, Holmes E, Lindon JC, et al (2010) Urinary metabolic phenotyping differentiates children with autism from their unaffected siblings and age-matched controls. J Proteome Res 9: 2996–3004. 20337404 10.1021/pr901188e

[14] Ming X, Stein TP, Barnes V, Rhodes N, Guo L (2012) Metabolic perturbance in autism spectrum disorders: a metabolomics study. J Proteome Res 11: 5856–5862. 23106572 10.1021/pr300910n

[15] Cai HL, Li HD, Yan XZ, Sun B, Zhang Q, et al (2012) Metabolomic analysis of biochemical changes in the plasma and urine of first-episode neuroleptic-naive schizophrenia patients after treatment with risperidone. J Proteome Res 11: 4338–4350. 22800120 10.1021/pr300459d

[16] Schicho R, Shaykhutdinov R, Ngo J, Nazyrova A, Schneider C, et al.. (2012) Quantitative Metabolomic Profiling of Serum, Plasma, and Urine by (1)H NMR Spectroscopy Discriminates between Patients with Inflammatory Bowel Disease and Healthy Individuals. J Proteome Res. 10.1021/pr300139q PMC3558013 22574726

[17] Soga T, Baran R, Suematsu M, Ueno Y, Ikeda S, et al (2006) Differential metabolomics reveals ophthalmic acid as an oxidative stress biomarker indicating hepatic glutathione consumption. J Biol Chem 281: 16768–16776. 16608839 10.1074/jbc.M601876200

[18] Sugimoto M, Hirayama A, Robert M, Abe S, Soga T, et al (2010) Prediction of metabolite identity from accurate mass, migration time prediction and isotopic pattern information in CE-TOFMS data. Electrophoresis 31: 2311–2318. 20568260 10.1002/elps.200900584

[19] Hirayama A, Kami K, Sugimoto M, Sugawara M, Toki N, et al (2009) Quantitative metabolome profiling of colon and stomach cancer microenvironment by capillary electrophoresis time-of-flight mass spectrometry. Cancer Res 69: 4918–4925. 19458066 10.1158/0008-5472.CAN-08-4806

[20] Soga T, Sugimoto M, Honma M, Mori M, Igarashi K, et al (2011) Serum metabolomics reveals gamma-glutamyl dipeptides as biomarkers for discrimination among different forms of liver disease. J Hepatol 55: 896–905. 21334394 10.1016/j.jhep.2011.01.031

[21] Yamasue H, Kuwabara H, Kawakubo Y, Kasai K (2009) Oxytocin, sexually dimorphic features of the social brain, and autism. Psychiatry Clin Neurosci 63: 129–140. 19335381 10.1111/j.1440-1819.2009.01944.x

[22] Baron-Cohen S, Lombardo MV, Auyeung B, Ashwin E, Chakrabarti B, et al (2011) Why are autism spectrum conditions more prevalent in males? PLoS Biol 9: e1001081. 21695109 10.1371/journal.pbio.1001081 PMC3114757

[23] Aoki Y, Abe O, Yahata N, Kuwabara H, Natsubori T, et al (2012) Absence of age-related prefrontal NAA change in adults with autism spectrum disorders. Transl Psychiatry 2: e178. 23092982 10.1038/tp.2012.108 PMC3565815

[24] Watanabe T, Yahata N, Abe O, Kuwabara H, Inoue H, et al (2012) Diminished medial prefrontal activity behind autistic social judgments of incongruent information. PLoS One 7: e39561. 22745788 10.1371/journal.pone.0039561 PMC3382122

[25] Lecavalier L, Aman MG, Scahill L, McDougle CJ, McCracken JT, et al (2006) Validity of the autism diagnostic interview-revised. Am J Ment Retard 111: 199–215. 16597187 10.1352/0895-8017(2006)111[199:VOTADI]2.0.CO;2

[26] Matsuoka K, Uno M, Kasai K, Koyama K, Kim Y (2006) Estimation of premorbid IQ in individuals with Alzheimer’s disease using Japanese ideographic script (Kanji) compound words: Japanese version of National Adult Reading Test. Psychiatry Clin Neurosci 60: 332–339. 16732750 10.1111/j.1440-1819.2006.01510.x

[27] Matsuoka K, Kim Y (2006) Japanese Adult Reading Test. Tokyo: Shinko-Igaku Publishers.

[28] Wechsler D (1981) Wechsler Adult Intelligence Scale-Revised. New York: Psychological Corporation.

[29] Wechsler D (1997) Wechsler Adult Intelligence Scale, 3rd edition. San Antonio: The Psychological Corporation.

[30] Sheehan DV, Lecrubier Y, Sheehan KH, Amorim P, Janavs J, et al.. (1998) The Mini-International Neuropsychiatric Interview (M.I.N.I.): the development and validation of a structured diagnostic psychiatric interview for DSM-IV and ICD-10. J Clin Psychiatry 59 Suppl 20: 22–33;quiz 34–57. 9881538

[31] Ooga T, Sato H, Nagashima A, Sasaki K, Tomita M, et al (2011) Metabolomic anatomy of an animal model revealing homeostatic imbalances in dyslipidaemia. Mol Biosyst 7: 1217–1223. 21258713 10.1039/c0mb00141d

[32] Baran R, Kochi H, Saito N, Suematsu M, Soga T, et al (2006) MathDAMP: a package for differential analysis of metabolite profiles. BMC Bioinformatics 7: 530. 17166258 10.1186/1471-2105-7-530 PMC1764210

[33] Ohashi Y, Hirayama A, Ishikawa T, Nakamura S, Shimizu K, et al (2008) Depiction of metabolome changes in histidine-starved Escherichia coli by CE-TOFMS. Mol Biosyst 4: 135–147. 18213407 10.1039/b714176a

[34] Johannesen JK, O’Donnell BF, Shekhar A, McGrew JH, Hetrick WP (2012) Diagnostic Specificity of Neurophysiological Endophenotypes in Schizophrenia and Bipolar Disorder. Schizophr Bull. 10.1093/schbul/sbs093 PMC3796068 22927673

[35] Delwing D, Bavaresco CS, Wyse AT (2008) Protective effect of nitric oxide synthase inhibition or antioxidants on brain oxidative damage caused by intracerebroventricular arginine administration. Brain Res 1193: 120–127. 18190896 10.1016/j.brainres.2007.11.052

[36] Zoroglu SS, Yurekli M, Meram I, Sogut S, Tutkun H, et al (2003) Pathophysiological role of nitric oxide and adrenomedullin in autism. Cell Biochem Funct 21: 55–60. 12579522 10.1002/cbf.989

[37] Shimmura C, Suda S, Tsuchiya KJ, Hashimoto K, Ohno K, et al (2011) Alteration of plasma glutamate and glutamine levels in children with high-functioning autism. PLoS One 6: e25340. 21998651 10.1371/journal.pone.0025340 PMC3187770

[38] Macciardi F, Lucca A, Catalano M, Marino C, Zanardi R, et al (1990) Amino acid patterns in schizophrenia: some new findings. Psychiatry Res 32: 63–70. 2161549 10.1016/0165-1781(90)90136-s

[39] Talkowski ME, Rosenfeld JA, Blumenthal I, Pillalamarri V, Chiang C, et al (2012) Sequencing chromosomal abnormalities reveals neurodevelopmental loci that confer risk across diagnostic boundaries. Cell 149: 525–537. 22521361 10.1016/j.cell.2012.03.028 PMC3340505

[40] Kim HW, Cho SC, Kim JW, Cho IH, Kim SA, et al (2009) Family-based association study between NOS-I and -IIA polymorphisms and autism spectrum disorders in Korean trios. Am J Med Genet B Neuropsychiatr Genet 150B: 300–306. 18563708 10.1002/ajmg.b.30798

[41] O’Donovan MC, Craddock N, Norton N, Williams H, Peirce T, et al (2008) Identification of loci associated with schizophrenia by genome-wide association and follow-up. Nat Genet 40: 1053–1055. 18677311 10.1038/ng.201

[42] Saransaari P, Oja SS (2000) Taurine and neural cell damage. Amino Acids 19: 509–526. 11140356 10.1007/s007260070003

[43] Moreno-Fuenmayor H, Borjas L, Arrieta A, Valera V, Socorro-Candanoza L (1996) Plasma excitatory amino acids in autism. Invest Clin 37: 113–128. 8718922

[44] Geier DA, Kern JK, Garver CR, Adams JB, Audhya T, et al (2009) A prospective study of transsulfuration biomarkers in autistic disorders. Neurochem Res 34: 386–393. 18612812 10.1007/s11064-008-9782-x

[45] Kern JK, Geier DA, Adams JB, Garver CR, Audhya T, et al (2011) A clinical trial of glutathione supplementation in autism spectrum disorders. Med Sci Monit 17: CR677–682. 22129897 10.12659/MSM.882125 PMC3628138

[46] Tu WJ, Chen H, He J (2012) Application of LC-MS/MS analysis of plasma amino acids profiles in children with autism. J Clin Biochem Nutr 51: 248–249. 23170055 10.3164/jcbn.12-45 PMC3491252

[47] Good P (2011) Does fever relieve autistic behavior by improving brain blood flow? Neuropsychol Rev 21: 66–67. 21249454 10.1007/s11065-011-9157-y

[48] Good P (2011) Do salt cravings in children with autistic disorders reveal low blood sodium depleting brain taurine and glutamine? Med Hypotheses 77: 1015–1021. 21925797 10.1016/j.mehy.2011.08.038

[49] Meister A (1991) Glutathione deficiency produced by inhibition of its synthesis, and its reversal; applications in research and therapy. Pharmacol Ther 51: 155–194. 1784629 10.1016/0163-7258(91)90076-x

[50] Pederzolli CD, Sgaravatti AM, Braum CA, Prestes CC, Zorzi GK, et al (2007) 5-Oxoproline reduces non-enzymatic antioxidant defenses in vitro in rat brain. Metab Brain Dis 22: 51–65. 17238006 10.1007/s11011-006-9041-2

[51] Pederzolli CD, Mescka CP, Zandona BR, de Moura Coelho D, Sgaravatti AM, et al (2010) Acute administration of 5-oxoproline induces oxidative damage to lipids and proteins and impairs antioxidant defenses in cerebral cortex and cerebellum of young rats. Metab Brain Dis 25: 145–154. 20431931 10.1007/s11011-010-9190-1

[52] Rossignol DA, Frye RE (2012) Mitochondrial dysfunction in autism spectrum disorders: a systematic review and meta-analysis. Mol Psychiatry 17: 290–314. 21263444 10.1038/mp.2010.136 PMC3285768

[53] Fillano JJ, Goldenthal MJ, Rhodes CH, Marin-Garcia J (2002) Mitochondrial dysfunction in patients with hypotonia, epilepsy, autism, and developmental delay: HEADD syndrome. J Child Neurol 17: 435–439. 12174964 10.1177/088307380201700607

[54] Filipek PA, Juranek J, Smith M, Mays LZ, Ramos ER, et al (2003) Mitochondrial dysfunction in autistic patients with 15q inverted duplication. Ann Neurol 53: 801–804. 12783428 10.1002/ana.10596

[55] Correia C, Coutinho AM, Diogo L, Grazina M, Marques C, et al (2006) Brief report: High frequency of biochemical markers for mitochondrial dysfunction in autism: no association with the mitochondrial aspartate/glutamate carrier SLC25A12 gene. J Autism Dev Disord 36: 1137–1140. 17151801 10.1007/s10803-006-0138-6

[56] Giulivi C, Zhang YF, Omanska-Klusek A, Ross-Inta C, Wong S, et al (2010) Mitochondrial dysfunction in autism. JAMA 304: 2389–2396. 21119085 10.1001/jama.2010.1706 PMC3915058

[57] Oliveira G, Diogo L, Grazina M, Garcia P, Ataide A, et al (2005) Mitochondrial dysfunction in autism spectrum disorders: a population-based study. Dev Med Child Neurol 47: 185–189. 15739723 10.1017/s0012162205000332

[58] Weiss LA, Shen Y, Korn JM, Arking DE, Miller DT, et al (2008) Association between microdeletion and microduplication at 16p11.2 and autism. N Engl J Med 358: 667–675. 18184952 10.1056/NEJMoa075974

[59] McCarthy SE, Makarov V, Kirov G, Addington AM, McClellan J, et al (2009) Microduplications of 16p11.2 are associated with schizophrenia. Nat Genet 41: 1223–1227. 19855392 10.1038/ng.474 PMC2951180

