# Supplementary material for: Altered Metabolites in the Plasma of Autism Spectrum Disorder: A Capillary Electrophoresis Time-of-Flight Mass Spectroscopy Study
Source: PLoS One. 2013 Sep 18;8(9):e73814. doi: 10.1371/journal.pone.0073814 (PMC3776798; doi:10.1371/journal.pone.0073814)
Supplement: Table S1 — Detected metabolites list. Metabolites detected in both the first and second sets, those in the first set only, and those in the second set only were listed respectively. (DOCX) [file pone.0073814.s001.docx]

| **Supplementary Table 1. Detected metabolites list** | | |  |  |
| --- | --- | --- | --- | --- |
| 118 metabolites both in the first and the second sets |  | 25 metabolites in the first set only |  | 23 metabolites in the second set only |
| 2-Aminobutyric acid |  | 1-Methyl-4-imidazoleacetic acid |  | 2-Aminoadipic acid |
| 2-Hydroxy-4-methylvaleric acid |  | 2-Hydroxyglutaric acid |  | 2-Oxoglutaric acid |
| 2-Hydroxybutyric acid |  | 3-Phosphoglyceric acid |  | 3-Hydroxypropionic acid |
| 2-Hydroxyvaleric acid |  | 10-Hydroxydecanoic acid |  | 4-Pyridoxic acid |
| 2-Oxobutyric acid |  | dGTP |  | 5-Hydroxylysine |
| 2-Oxoisovaleric acid |  | Diphenylcarbazide |  | Alliin |
| 2-Oxooctanoic acid |  | Fumaric acid |  | AMP |
| 3-Aminobutyric acid |  | GABA |  | Butyrylcarnitine |
| 3-Aminoisobutyric acid |  | Glucaric acid |  | Cholic acid |
| 3-Hydroxybutyric acid |  | Glutaric acid |  | Glucuronic acid |
| 3-Indoxylsulfuric acid |  | Glutathione (GSSG)_divalent |  | Guanosine |
| 3-Methylhistidine |  | Hypotaurine |  | Hippuric acid |
| 4-Aminoindole |  | Imidazolelactic acid |  | Ibuprofen |
| 4-Methyl-2-oxovaleric acid 3-Methyl-2-oxovaleric acid |  | N6-Acetyllysine |  | Inosine |
| 4-Oxovaleric acid |  | N-Acetylaspartic acid |  | Isobutylamine |
| 5-Aminoindole |  | N-Acetylleucine |  | Isobutyrylcarnitine |
| 5-Oxohexanoic acid |  | N-Ethylglycine |  | m-Toluic acid |
| 5-Oxoproline |  | Phosphocreatine |  | Mucic acid |
| 6-Hydroxyhexanoic acid |  | Phosphorylcholine |  | Octanoylcarnitine |
| 8-Hydroxyoctanoic acid |  | Ser-Glu |  | Ribulose 5-phosphate |
| Acetoacetic acid |  | Serotonin |  | S-Sulfocysteine |
| ADMA |  | Sulfotyrosine |  | Tartaric acid |
| ADP |  | Terephthalic acid |  | Theobromine |
| Ala |  | trans-Glutaconic acid |  |  |
| Arg |  | Urocanic acid |  |  |
| Asn |  |  |  |  |
| Asp |  |  |  |  |
| ATP |  |  |  |  |
| Benzoic acid |  |  |  |  |
| Betaine |  |  |  |  |
| Betonicine |  |  |  |  |
| Butyric acid |  |  |  |  |
| Caffeine |  |  |  |  |
| Carnitine |  |  |  |  |
| Choline |  |  |  |  |
| cis-Aconitic acid |  |  |  |  |
| Citric acid |  |  |  |  |
| Citrulline |  |  |  |  |
| Creatine |  |  |  |  |
| Creatinine |  |  |  |  |
| Cyclohexanecarboxylic acid |  |  |  |  |
| Cyclohexylamine |  |  |  |  |
| Cysteine glutathione disulfide |  |  |  |  |
| Cystine |  |  |  |  |
| Decanoic acid |  |  |  |  |
| Diethanolamine |  |  |  |  |
| Dyphylline |  |  |  |  |
| Ethanolamine |  |  |  |  |
| Ethanolamine phosphate |  |  |  |  |
| Gln |  |  |  |  |
| Glu |  |  |  |  |
| Gluconic acid |  |  |  |  |
| Gly |  |  |  |  |
| Glyceric acid |  |  |  |  |
| Glycerol 3-phosphate |  |  |  |  |
| Glycerophosphocholine |  |  |  |  |
| Glycocholic acid |  |  |  |  |
| Glycolic acid |  |  |  |  |
| Glyoxylic acid |  |  |  |  |
| Guanidoacetic acid |  |  |  |  |
| Heptanoic acid |  |  |  |  |
| Hexanoic acid |  |  |  |  |
| His |  |  |  |  |
| Hydroxyproline |  |  |  |  |
| Hypoxanthine |  |  |  |  |
| Ile |  |  |  |  |
| Indole-3-acetic acid |  |  |  |  |
| Isethionic acid |  |  |  |  |
| Isocitric acid |  |  |  |  |
| Kynurenine |  |  |  |  |
| Lactic acid |  |  |  |  |
| Lauric acid |  |  |  |  |
| Leu |  |  |  |  |
| Lys |  |  |  |  |
| Malic acid |  |  |  |  |
| Met |  |  |  |  |
| Methionine sulfoxide |  |  |  |  |
| Myristoleic acid |  |  |  |  |
| N,N-Dimethylglycine |  |  |  |  |
| N2-Phenylacetylglutamine |  |  |  |  |
| N5-Ethylglutamine |  |  |  |  |
| N6-Methyllysine |  |  |  |  |
| N-Acetyl-β-alanine |  |  |  |  |
| N-Methylproline |  |  |  |  |
| O-Acetylcarnitine |  |  |  |  |
| Octanoic acid |  |  |  |  |
| Ornithine |  |  |  |  |
| Pelargonic acid |  |  |  |  |
| Perillic acid |  |  |  |  |
| Phe |  |  |  |  |
| Pipecolic acid |  |  |  |  |
| Piperidine |  |  |  |  |
| Pro |  |  |  |  |
| Propionic acid |  |  |  |  |
| Pyruvic acid |  |  |  |  |
| Quinic acid |  |  |  |  |
| Sarcosine |  |  |  |  |
| SDMA |  |  |  |  |
| Ser |  |  |  |  |
| S-Methylcysteine |  |  |  |  |
| Stachydrine |  |  |  |  |
| Succinic acid |  |  |  |  |
| Taurine |  |  |  |  |
| Thiaproline |  |  |  |  |
| Thr |  |  |  |  |
| Tiglic acid |  |  |  |  |
| Trigonelline |  |  |  |  |
| Trimethylamine N-oxide |  |  |  |  |
| Trp |  |  |  |  |
| Tyr |  |  |  |  |
| Undecanoic acid |  |  |  |  |
| Urea |  |  |  |  |
| Uric acid |  |  |  |  |
| Uridine |  |  |  |  |
| Val |  |  |  |  |
| Valeric acid |  |  |  |  |
| β-Ala |  |  |  |  |
| γ-Butyrobetaine |  |  |  |  |
